# The Effect of Schedule Thinning on Student Behavior During the Caught Being Good Game

**DOI:** 10.1177/01454455221129993

**Published:** 2022-11-13

**Authors:** Clare Bohan, Sinéad Smyth

**Affiliations:** 1 Dublin City University, Ireland

**Keywords:** academic engagement, Caught Being Good Game, classroom management, disruptive behavior, positive behavior intervention

## Abstract

The Caught Being Good Game (CBGG) is a classroom management intervention which is described as a variation of the classic Good Behavior Game (GBG). It is based on the principle of positive reinforcement, such that teams of students can earn points for following the class rules during the game. Points are awarded by the teacher at different intervals during the game and these intervals were the focus of the current study. We aimed to determine if the CBGG is effective with an initially dense schedule of reinforcement which is progressively thinned. The efficacy of the CBGG in targeting academic engagement and disruptive behavior was demonstrated for one primary school class and for two target students in that class. The game remained effective when the reinforcement schedule was thinned from 2 minutes, up to 5 minutes. This has potential implications for teacher time saving while playing the game.

## Introduction

Behavioral classroom management approaches often incorporate the principle of positive reinforcement which serves as a key mechanism in promoting future engagement in desirable behavior. Positive reinforcement is a common feature in praise interventions (Moore et al., 2019), token economies (Maggin et al., 2011) and group contingency interventions (Maggin et al., 2012, 2017). The Good Behavior Game (GBG; Barrish et al., 1969) is one such group contingency intervention which has been effective in promoting academic engagement and reducing disruptive behavior in classrooms (e.g., Tanol et al., 2010; Wright & McCurdy, 2012). In this game-based intervention, students are placed on teams, and teams on which a member breaks a class rule receive a mark. Teams remaining under a certain criterion of marks at the end of the game are eligible for a reward. Thus, the game functions as an interdependent group contingency: the same contingencies are in place for all students, however it is the performance of the group that determines whether a reward is obtained (Litow & Pumroy, 1975). Although positive reinforcement is incorporated in the GBG, the behavioral principles underlying the intervention have been interpreted in different ways. For example, some note that the GBG incorporates positive punishment on a fixed ratio (FR 1; Wright & McCurdy, 2012), such that every time a class rule is violated, a mark is given to a team, serving as a punisher. It has also been recognised however, that a teacher likely does not actually observe every single incident of misbehavior which occurs during a class (Wright & McCurdy, 2012), thereby creating a variable ratio (VR) schedule of punishment (Joslyn & Vollmer, 2020). The GBG has also been described as incorporating positive reinforcement in the form of differential reinforcement of low rates of disruptive behavior (DRL; Mitchell et al., 2015; Wright & McCurdy, 2012), such that students are rewarded for displaying low rates of disruption across the game period.

Recent developments in GBG literature have seen a surge in research on a positive version of the GBG, often termed the Caught Being Good Game (CBGG) by researchers (e.g., Bohan et al., 2021; Wahl et al., 2016; Wright & McCurdy, 2012). This game omits the fixed or variable ratio of punishment and instead, provides teams of students with points (i.e., positive reinforcement) at different intervals throughout the class. This procedure has been termed differential reinforcement of other behavior (DRO), such that reinforcement is delivered at intervals when behavior other than disruption is displayed by teams of students (Wright & McCurdy, 2012). Teams are then eligible for a prize if they surpass a criterion of points at the end of the game. It has been suggested that the type of reinforcement applied during the CBGG is a fixed or variable momentary DRO schedule, depending on how exactly points are awarded (FM-DRO/VM-DRO; Wright & McCurdy, 2012). Other researchers have used a fixed whole interval DRO schedule (e.g., Groves & Austin, 2020). This is a schedule whereby reinforcement is contingent on the absence of problem behavior during a whole preceding interval. Both types of schedule have been effective during the CBGG. The CBGG has been effective in targeting academic engagement and disruption across primary (e.g., Bohan & Smyth, 2022; Groves & Austin, 2020; Wahl et al., 2016; Wright & McCurdy, 2012) and secondary school classrooms (e.g., Bohan et al., 2021; Ford et al., 2022), while maintaining similar effectiveness to the GBG in comparison studies (Wahl et al., 2016; Wright & McCurdy, 2012).

Previous studies on the CBGG have set schedules of reinforcement for the duration of the study without changing the schedule over time. For example, Wright and McCurdy (2012) used a VI 4 min momentary DRO schedule for behavior checks and this was not changed/lengthened throughout the CBGG phases despite behavior improving across the classes. Other studies have used even denser schedules. For example, when examining the efficacy of the CBGG with a high school class, Ford et al., (2022) prompted the teacher every 2 minutes to scan the classroom. The teacher was encouraged to scan the room between 0 and 30 seconds after the prompt to ensure there was some variability in the schedule, however the schedule remained dense throughout the study. Bohan and Smyth (2022) recently implemented the CBGG with a middle primary school class in an Irish primary school, setting quite dense intervals of 2 minutes between points. The game was effective in targeting academic engagement and disruption across the whole class as well as with two individual target students. The decision making process behind these interval lengths is often not described, however it is likely that researchers take teacher considerations into account where possible. For example, Bohan et al. (2021) describe the researcher and teacher deciding upon a 5 minute interval for CBGG implementation to allow for minimal distraction for the teacher. Indeed, similar patterns are observed in the GBG literature where many studies have examined the intervention, yet few manipulated the schedule of punishment in place during the game. One exception is a recent study by Wiskow et al. (2021) where a “modified” GBG was compared to the traditional GBG and the CBGG. The modified GBG involved a variable interval schedule of punishment whereby the intervention agent would conduct a behavior check and give marks to teams where a member was breaking a class rule at that time. This random and variable interval was between 1 and 4 minutes throughout the study, with no period of systematic interval lengthening.

Schedule thinning has been applied systematically in the past to fade out interventions and to make them less time intensive for the intervention agent. Benefits of decreasing intervention intensity include enhancing the ease of management of reinforcement delivery for the intervention agent and making the intervention more naturalistic and therefore potentially more generalizable (LeBlanc et al., 2002). In the classroom management literature, particularly that focused on the popular GBG/CBGG, interventions are often put in place and kept in place throughout a study without allowing for a period of schedule thinning or intervention fading. This means that teachers or other intervention agents are potentially implementing an intervention at a level much more intense than needed, which depletes valuable resources. One exception was a study by Conklin et al. (2017), which evaluated Class-Wide Function-related Intervention Teams (CW-FIT), a package intervention which incorporates a group contingency game very similar to the CBGG. In this study, the authors did incorporate a schedule thinning procedure whereby the teacher began by awarding points to teams every 1 to 2 minutes and thinned the schedule to every 3 to 5 minutes over time as students became more proficient at prosocial skills taught as part of the intervention. The authors did not go into detail on the schedule thinning procedures, nor did they specify at what point schedule thinning occurred so that effectiveness of the procedure can be analysed. Naylor et al. (2018) conducted a similar study on the CW-FIT, in which they stated schedule thinning was incorporated into the group contingency procedure. Similarly to Conklin et al. (2017), they did not outline at what stage they began to thin the schedule, simply that they began with a dense schedule (1–2 minutes) and progressed to a leaner schedule (1–4 minutes). Similarly, within the GBG research, to our knowledge, there is no example of the schedule of punishment being thinned over time in response to improvements in behavior. A potential reason for this, is that the schedule of punishment is thinned naturally as disruptive behaviour decreases during the GBG. Less disruptive behavior means there are less opportunities for the intervention agent to administer marks. This again highlights the fundamental differences between the principles underlying the GBG and CBGG because such natural schedule thinning cannot take place during the CBGG but must instead be planned and implemented.

No study on the CBGG has explicitly examined the effect of changing the schedule of reinforcement over time despite recent calls for evaluations of procedural variations of the game (Joslyn et al., 2019). An application of a schedule thinning procedure during the CBGG has the potential to occupy a distinct and important gap in the literature and has the capacity to assist in understanding the mechanisms surrounding the effectiveness of the CBGG as a distinct intervention. It can be distinguished from the traditional GBG in this context given that the schedule of reinforcement has the potential for manipulation during the CBGG in a way that cannot be established in the GBG. Published studies on similar game-based interventions have changed the schedule of reinforcement over time (e.g., Conklin et al., 2017; Naylor et al., 2018), however detailed accounts of the timing of the schedule changes are not provided. Providing a detailed account of the steps taken during the schedule thinning procedure is important to inform practice, as behavior analytic applications should be technological, that is, the techniques applied in a study should be identified and described completely (Baer et al., 1968). There is therefore scope to examine the CBGG, beginning with a dense schedule of reinforcement and progressing to a thinner schedule over time.

### The Current Study

The current study aimed to address the aforementioned gap in the literature by evaluating the CBGG in an Irish primary school class, progressively thinning the schedule of opportunities for reinforcement. To maximise our understanding of the game’s effects, data were collected on the whole class, and also on two teacher-selected target students. This allowed for the potential identification of non-responders to the intervention (Bohan & Smyth, 2022; Donaldson et al., 2017). Furthermore, the study aimed to assert whether the part-taking teacher and students found the CBGG to be socially valid and acceptable, an important consideration in any behavior analytic research (Wolf, 1978).

## Method

### Participants and Setting

Participants were recruited from a primary school in a densely populated, urban area of Dublin, Ireland. Twenty-six students (15 female, 11 male) in a senior infants class took part. Senior infants is the second year of formal schooling in the Irish school system and is approximately equivalent to Kindergarten in the USA school system. The class teacher, Ms. Leonard (pseudonym), was a 48-year-old female with 12 years of teaching experience. She responded to a recruitment call for teachers who were willing to try a new classroom management intervention and did not have previous experience with implementing the CBGG. Ms. Leonard read about the study and signed a consent form before the study commenced. Ms. Leonard chose two students for individual behavior monitoring based on her evaluation of class behavior, such that she was asked to choose students for whom she had specific concerns regarding their behavior. Ellie was a 5-year-old female student and Katie was a 6-year-old female student (pseudonyms). Parents of all participating students completed consent forms and students completed assent forms before the study commenced. The study obtained full ethical approval from the relevant university’s research ethics committee.

### Materials

The teacher was provided with a laminated copy of the game rules and five scoreboards, one for each table in the classroom. The scoreboards were colored penguins with spaces for points to be colored on when administered. Each penguin was color-coded to correspond with a table, as each of the five tables in the room had an assigned color. The teacher was given an Octopus watch (version 1) which was pre-programmed to vibrate and remind her to scan the classroom intermittently, depending on the current schedule. The Octopus watch could be pre-programmed to vibrate at regular intervals. Ms. Leonard chose the prize for the CBGG based on what she had used with students before. Prizes used were stamps and stickers, which were cost effective and appeared desirable to the students based on the teacher’s evaluation. The teacher stuck a checklist to the wall which outlined the steps of the game. Observers used the same checklist to carry out treatment integrity checks. Data were collected by observers with paper and pen and interval changes were signalled via earphones plugged in to a smart phone.

### Dependent Variables

Data were collected on academically engaged behavior (AEB) and disruptive behavior (DB). AEB was defined as a student giving their attention to the academic task ongoing, which included writing, coloring, reading aloud or to oneself, conversing with a peer about the task (where this has been permitted by the teacher), eye contact was oriented toward the task or teacher or the student was using the class sharpener or walking in the direction of the sharpener at the time of recording. A student was not considered to be engaged if engaging in any of the outlined disruptive behaviors. DB was measured across the three categories of verbal disruption (VD), out-of-seat behavior (OOS), and motor disruption (MD). VD occurred when a student engaged in any vocalization not authorized by the teacher and unrelated to the work ongoing in the classroom. This included singing, whistling, humming, and shouting out. OOS was defined as a student leaving their seat and moving more than 1 metre from their chair, with the exception of a student leaving their chair, walking directly to the class pencil sharpener and back to their chair. MD occurred when a student was playing with an object in a manner incompatible with the academic task, turning in one’s chair away from the task for >3 seconds, leaving one’s head on the desk, swinging on two legs of the chair or physically interacting with a peer in a manner which is incompatible with the academic task. The three subcategories of DB were combined as one composite DB variable for the purposes of analysis.

### Data Collection and Interobserver Agreement

Data were collected up to four times per week in the classroom during the last period of the school day. Data collection sessions lasted 20 minutes and during this period, the class engaged in a mathematics lesson. The session involved the teacher explaining a concept on the board for 4 to 5 minutes followed by independent seatwork for 15 to 20 minutes. Data were collected via momentary time sampling (AEB) and partial interval recording (DB), in 10 second intervals with 5 seconds between intervals to record. DB was a discrete and countable behaviour, however given the large number of children in the class, and observer resources, partial interval recording was deemed appropriate. Momentary time sampling was selected for AEB as this was not a discrete behavior, and again resources were not sufficient to continuously monitor the behaviour (LeBlanc et al., 2016). Students who were not designated target students, were observed one at a time in a fixed order in order to obtain a measure of class-wide behavior. In every second interval, one of two target students was observed. This meant that in a 20 minute observation session, there were 80 intervals. Each target student would be observed for 20 intervals each and a general member of the class in the remaining 40 intervals.

Second observers were trained undergraduate and postgraduate psychology students who volunteered to assist with data collection for the purposes of collecting interobserver agreement (IOA) data. IOA data were collected on 38.46% of observation sessions overall and during at least 20% of baseline and intervention phases, as per What Works Clearinghouse (WWC) recommendations (WWC, 2020). It was collected at least once per phase for each outcome. IOA was collected on 38.46% of occasions for the whole class, 33.3% of occasions for Ellie and 37.5% of occasions for Katie. IOA for student behavior was calculated using interval-by-interval agreement (Cooper et al., 2020) and dividing the number of agreements by the total number of observation intervals and multiplying by 100 to obtain a percentage. Although calculating the rate of agreement for observations conducted using discontinuous methods may yield inflated levels of interobserver agreement (e.g., Rapp et al., 2011), the data collection methods were deemed appropriate based on resources and recommendations in the literature (LeBlanc et al., 2016). Mean IOA for AEB for the whole class was 87.4% (range = 70.3%–97.5%), for Ellie was 84.6% (range = 80%–90%) and for Katie was 83.1% (range = 75.3%–95.5%). Mean IOA for DB for the whole class was 92.75% (range = 88.3%–95%), for Ellie was 90.3% (range = 85%–94.1%) and for Katie was 89.7% (range = 80%–97.9%).

### Experimental Design

An ABAB reversal design was used to evaluate the CBGG across the class and individual target students taking part (A = baseline, B = CBGG). Across the second CBGG phase, schedule thinning was introduced, whereby the schedule of reinforcement was thinned from 2 minutes, to 3 minutes, to 4 minutes to 5 minutes over a series of observation sessions. Phase change decisions were determined a priori based on the teacher’s schedule and timeframe in which the study was to be conducted, however some flexibility allowed for decisions to be made based on the classwide behavior.

### Procedure

#### Baseline

During baseline, the teacher proceeded with planned educational tasks with no intervention in place. There was no specific contingency in place for rewarding positive behavior during educational tasks and disruptive behavior was addressed with verbal warnings. During the last day of baseline data collection, the teacher was asked to covertly assign team points, without letting the students know about it to allow her to get used to the watch vibrations, practice the game procedures privately and to assist with setting a points criterion for intervention phases.

#### Teacher training

Teacher training took place during one session after school the day before baseline data collection finished. Ms. Leonard was shown the behavioral data for her class and for the individual target students within the class. She was then talked through the CBGG procedures with the aid of a PowerPoint presentation. There was an opportunity to ask questions and Ms. Leonard assisted with choosing an appropriate reinforcer to use during the game (i.e., stamps and stickers, as outlined earlier). Ms. Leonard was introduced to the concept of schedule thinning. She was trained in implementation of the CBGG with 2 minute intervals between behavior checks. She was told that short meetings introducing her to thinned schedules would take place before each schedule change.

#### Intervention: Caught being good game

The CBGG was introduced to the class during the first session following completion of baseline data collection. The class was divided into five teams which were color coded based on their table, that is, the Red team, the Blue team, etc. The teacher told the children about the game, introducing it as the “Penguin game,” to tie in with the fact that the scoreboards were created with a penguin theme. Students were told that during the game there would be 10 chances to earn a point for following the class rules. These rules were as follows: *Look at and Listen to your teacher; Hands up and wait for your teacher; Do your best at your work; Respect your friends & let them do their work; Stay in your seat*. During the initial 2 minute version of the CBGG, the game would take place for 20 minutes.

Ms. Leonard announced that the game had started after explaining how the game was played and reviewing the class rules. When prompted to scan the room and award points if appropriate, Ms. Leonard would do so on the corresponding penguin scoreboard for each team, by coloring in 1 of 10 buttons on the penguins’ torso. The goal for the 2 minute version of the game was 7 points (out of a possible 10). This criterion was calculated by taking the average amount of points earned by each team during the last day of baseline and adding 10%. Similar protocols have been followed in previous research (e.g., Bohan & Smyth, 2022; Ford et al., 2022).

Prizes were awarded daily immediately after the game had ended. Teams meeting or exceeding the 7-point goal were eligible to receive the prize. The prize was a choice between one of two stamps, or a sticker. The stamps were brightly colored markers which would make different shaped and colored stamps, for example, a red apple, an orange star. The choice given daily was varied in that the teacher chose 2 different markers from a set of 10. The team with the most points got to choose their stamp or sticker first.

#### Schedule thinning

In the second CBGG phase, the schedule of reinforcement was thinned over time. The intervention was applied at an intensive level to begin with, with 2 minutes between reinforcement opportunities. It was decided to increase the time between behavior checks in 1 minute increments. Although this is a large percentage change in the schedule (an increase of 50% initially from 2 to 3 minutes, 33.33% increase from 3 to 4 minutes, and 25% increase from 4 to 5 minutes), it is still a short time frame and was deemed easy to understand and to remember for the teacher implementing the intervention. The terminal goal was 5 minutes. Table 1 summarizes the phases, maximum points available in each phase, and points criterion for each phase.

**Table 1.** Description of Intervention Phases Across the Study.

Phase^a^	Length of interval	Maximum points available	Criterion
B1	2 minutes	10	7
B2	2 minutes	10	7
B2	3 minutes	7	5
B2	4 minutes	5	3
B2	5 minutes	4	3

a B1 and B2 refer to the intervention phases, with 2 minute intervals between opportunities for points. In phase B2, the schedule is thinned as outlined in the table.

### Treatment Integrity

Ms. Leonard stuck a treatment integrity checklist to her classroom wall to serve as a prompt to complete each step of the CBGG. Treatment integrity data was collected during 100% of intervention data collection sessions. If treatment integrity dropped below 80% for more than 1 day in a row, this was brought to the teacher’s attention via email or in person and she was encouraged to use her checklist and complete each step. Overall mean treatment integrity across all intervention phases was 76.1% (range = 45.5%–100%).

### Social Validity

Following the final day of data collection, the teacher and students completed social validity measures. Ms. Leonard completed the Behavior Intervention Rating Scale (BIRS; Elliott & Von Brock Treuting, 1991) and students completed a modified version of the Children’s Intervention Rating Profile (CIRP; Mitchell et al., 2015; Witt & Elliott, 1985). The BIRS was modified to refer to the intervention in the past tense and to refer to “students”. Ms. Leonard was asked to answer the BIRS questions with reference to the CBGG generally. A number of additional, open-ended questions were added as follows: “*Did you have a preference for any particular version of the CBGG (2, 3, 4 or 5 minutes between points)?*”; “*Did you think any particular version was more/less effective than another?*”; “*Did you think any particular version was easier/more desirable to implement than others?*”; “*Do you have any further comments/feedback on the CBGG?*.”

The modified CIRP is a social validity measure with eight items such as “Did you like participating in the game?,” to which students answered “yes” or “no.” The measure was similar to that used in recent studies on the GBG and CBGG by Mitchell et al. (2015) and Bohan et al. (2021), however more substantial modifications were made for use in this study to make it suitable for the senior infants population. This included the rephrasing of some questions and including smiley faces and thumbs up/down as options to indicate approval/disapproval. The highest rating a student could give the game was eight. If a student responded negatively to a question, the researcher would ask them why and write down their responses.

### Data Analysis

Initially, the study design was evaluated taking guidance from the WWC design standards (WWC, 2020). Graphed data was analyzed visually, considering level, trend and variability in the data, as well as immediacy of effect and rate of overlap between phases. Effect sizes were calculated using Tarlow’s recommendations for calculation of Tau and Tau calculator (Tarlow, 2016, 2017). Tau effect sizes were calculated for the first four phases and weighted mean effect sizes were subsequently calculated for each participant and outcome. Tau values were not calculated for the schedule thinning phases (i.e., the CBGG with intervals of more than 2 minutes) as data variability across these phases was expected to be low and therefore it was deemed not appropriate. Vannest and Ninci (2015) suggest interpreting Tau values of .20 as a small effect, .20 to .60 as a moderate effect, .60 to .80 as a large effect, and .80+ as a very large effect.

## Results

### WWC Design Standards

The study design was in line with the WWC design standards as far as possible (WWC, 2020). The CBGG (i.e., the independent variable) was systematically manipulated throughout the study. IOA data were collected at least 20% of the time overall and in each condition, and at least once per phase for each outcome. There were at least three attempts to demonstrate intervention effects. The WWC (2020) asserts that phases should have a minimum of three data points to meet the design standards with reservations and more than five per phase to meet the standards fully. Our first two phases had 4 data points per phase, therefore we consider the study design to meet the WWC standards with reservations.

### Visual Analyses

#### Class-wide effects

**Figure 1.**
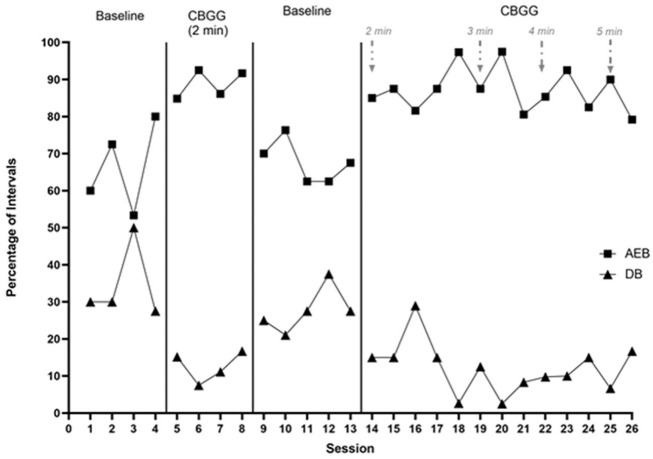
Percentage of intervals with academically engaged behavior (AEB) and disruptive behavior (DB) across study phases for Ms. Leonard’s class group. *Note.* The arrows specify points where the schedule is modified.

Data on class-wide AEB and DB across phases are presented in Figure 1. During the first baseline phase, AEB occurred during a mean of 66.5% of intervals (range = 53.3%–80%). AEB was highly variable during this phase. Class-wide DB occurred at a high, stable rate (*M* = 34.4%, range = 27.5%–50%), with one data point noticeably exceeding the other three (data point 3; see Figure 1). When the CBGG was introduced, AEB increased immediately and stabilised across the phase (*M* = 88.8%, range = 84.9%–92.5%). There was no overlap between this intervention phase and the preceding baseline phase. DB reduced immediately upon introduction of the CBGG (*M* = 12.6%, range = 7.5%–16.7%). This reflects a large reduction and there was no overlap here with the initial baseline phase.

The CBGG was withdrawn in the following phase and AEB decreased immediately and substantially (*M* = 67.8%, range = 62.5%–76.3%). There was no overlap between this phase and the intervention phase which preceded it. AEB remained low and quite stable across the phase with no obvious trend in the data. There was an immediate and moderate increase in DB (*M* = 27.7%, range = 21.1%–37.5%). There was a steady increase across most of the phase and no overlap with the previous intervention phase. When the CBGG was reinstated, AEB increased substantially (*M* = 87.8%, range = 81.6%–97.4%). There was no overlap with the previous withdrawal phase. There was an immediate decrease in DB (*M* = 15.3%, range = 2.6%–28.95%). There was one data point here where DB occurred at a rate similar to baseline phases (data point 16; see Figure 1). It was evident that a high rate of verbal disruption took place during that observation session.

When the schedule was thinned to 3 minutes, AEB remained high (*M* = 88.5%, range = 80.6%–97.5%), particularly across the first two data points. There was a decrease during the third data point under this modification, however AEB did not decrease enough to overlap with either of the baseline phases. DB remained low when this adaptation was made (*M* = 7.8%, range = 2.5%–12.5%). With 4 minutes between opportunities to earn a point, AEB remained high and stable (*M* = 86.8%, range = 82.5%–92.5%), and DB remained low and stable (*M* = 11.6%, range = 9.8%–15%). Finally, as the schedule was thinned to 5 minutes, the CBGG was implemented across two sessions. AEB remained high during the first data point in this phase, however decreased during the second data point (*M* = 84.6%, range = 79.2%–90%). Treatment integrity was low at data point 26 (45.5%) and it was the only intervention data point across all phases to overlap with any baseline data point. DB occurred at a low rate and was stable when the CBGG was played with 5 minutes intervals (*M* = 11.7%, range = 6.7%–16.7%).

#### Target Students

**Figure 2.**
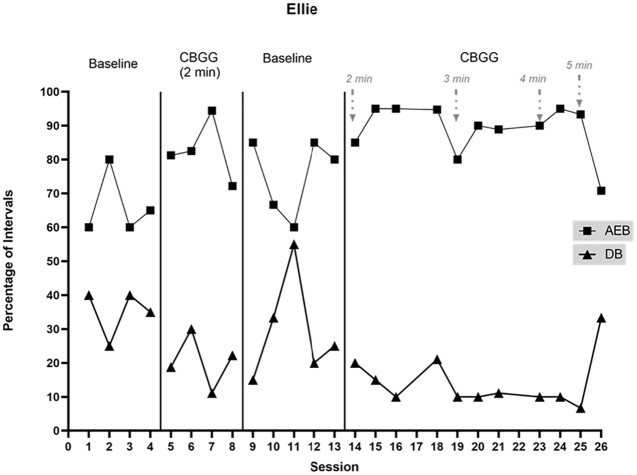
Percentage of intervals with academically engaged behavior (AEB) and disruptive behavior (DB) across study phases for Ellie. *Note.* The arrows specify points where the schedule is modified.

**Figure 3.**
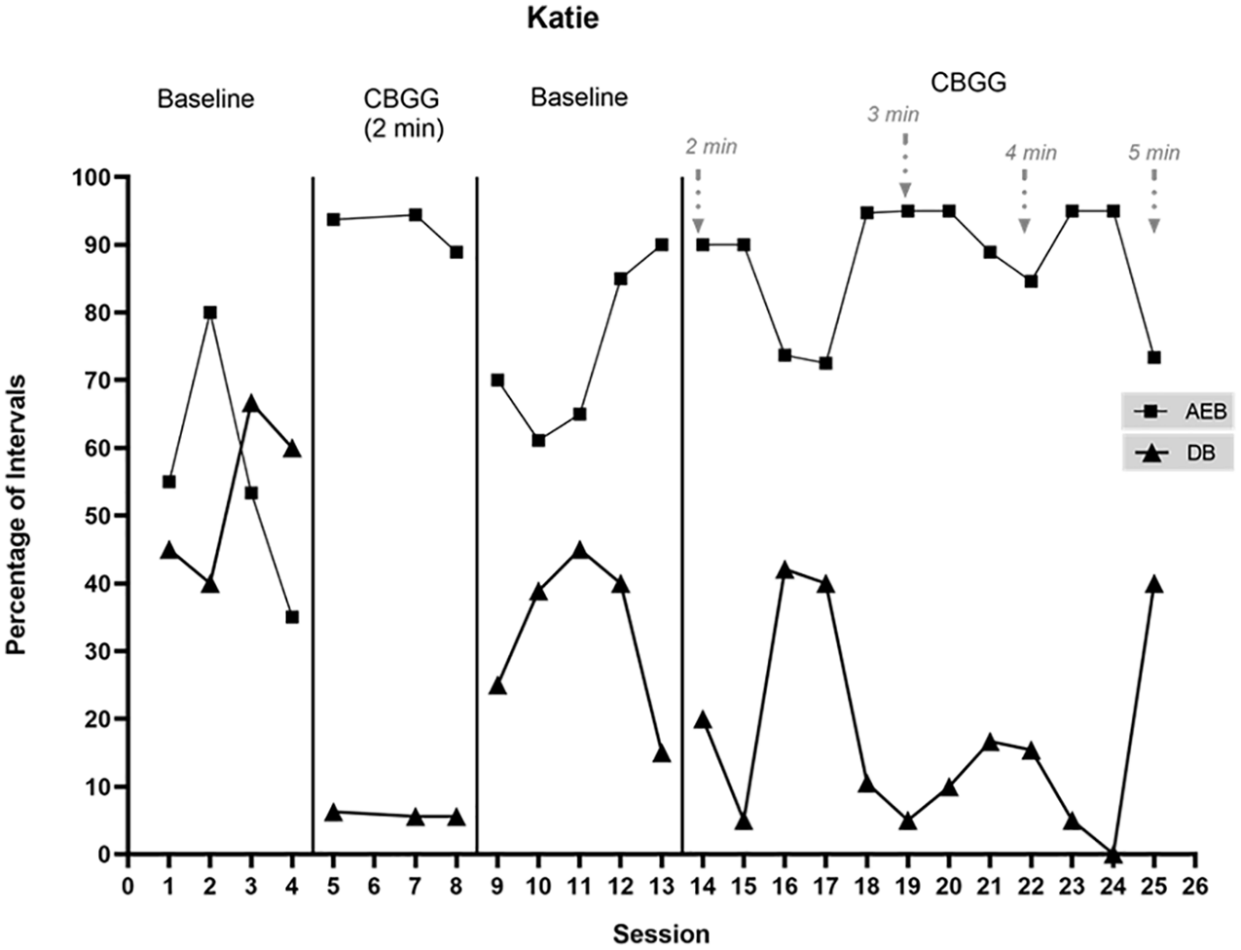
Percentage of intervals with academically engaged behavior (AEB) and disruptive behavior (DB) across study phases for Katie. *Note.* The arrows specify points where the schedule is modified.

Data on AEB and DB for Ellie and Katie are presented in Figures 2 and 3.

##### Ellie

At baseline, Ellie’s rate of AEB followed a similar pattern to the whole class in that it was variable and generally low (*M* = 66.3%, range = 60%–80%). Ellie’s overall rate of DB was high at baseline (*M* = 35%, range = 25%–40%) and very similar to the whole class DB. When the CBGG was introduced, Ellie’s AEB increased immediately and substantially and was sustained across three data points. AEB decreased again towards the end of the phase (*M* = 82.6%, range = 72.2%–94.4%), in contrast to the whole class AEB which remained high. The final data point in this phase overlapped with one data point in the baseline phase. There was an immediate decrease in DB initially however data were variable and the second data point in this phase overlapped with the baseline phase, before DB decreased again (*M* = 20.5%, range = 11.11%–30%). Ellie’s DB was higher than that of the whole class during this phase.

During the first withdrawal phase, Ellie’s AEB was highly variable and was not similar to the class, but despite this variability, her AEB was higher than the whole class on average (*M* = 75.3%, range = 60%–85%). The overall level of AEB remained lower than during the previous intervention phase, however there was a high degree of overlap. Despite an initial decrease in DB when the game was withdrawn, Ellie’s DB increased sharply toward the middle of phase, before dropping again (*M* = 29.7%, range = 15%–55%). Data were variable throughout this phase, however mean DB was similar to that of the class. When the CBGG was reintroduced, there was an increase in Ellie’s AEB (*M* = 92.4%, range = 85%–95%). Again, her level of AEB was higher than that of the class and just one of the four data points in this phase overlapped with the preceding baseline phase. DB stabilised during this phase and returned to levels more similar to the previous intervention phase (*M* = 16.5%, range = 10%–21.1%).

With the initial adaptation to the reinforcement schedule (3 minutes), Ellie’s AEB occurred during a mean of 86.3% of intervals (range = 80%–90%), which demonstrates a slight decrease in level when compared to the previous 2 minute schedule. Behavior was similar to the whole class here. DB remained low and stable (*M* = 10.4%, range = 10%–11.1%). With 4 minutes between reinforcement opportunities, AEB was high across the two sessions for which Ellie was present (*M* = 92.5%, range = 90%–95%) and DB remained low (*M* = 10%). With the schedule thinned to 5 minutes, AEB was variable, occurring during a mean of 82.1% of intervals (range = 70.8%–93.3%). The low rate of AEB during the final data point corresponds with low AEB across the class and low treatment integrity by the teacher. DB was initially low but increased to a very high level (*M* = 20%, range = 6.7%–33.3%). This coincided with a low level of treatment integrity during the final data point.

##### Katie

Katie’s rate of AEB was variable across the initial baseline phase, with a decreasing trend across the final three data points (*M* = 55.8%, range = 35%–80%). Her mean level of AEB was much lower than that of the whole class. DB was very high during this phase and always occurred at a higher rate than the class as a whole (*M* = 52.9%, range = 40%–66.7%). An immediate and large increase in AEB occurred when the CBGG was introduced (*M* = 92.4%, range = 88.9%–94.4%), such that Katie’s AEB was higher than the whole class mean for this phase. Katie’s DB decreased immediately and substantially (*M* = 5.8%, range = 5.6%–6.3%) and was lower than that of the whole class. There was no overlap with the initial baseline phase. It must be noted here that at the beginning of this CBGG phase, Katie moved seats to the other side of her table. When the game was introduced, this move was requested by her classmate who she spoke to a lot (at another close by table). Katie agreed to it, agreeing that it would help her earn more points. Ms. Leonard allowed this seat change upon the students’ request. Although this change of seats serves as a confounding variable, it came about as a direct result of the game being introduced, at the request of the students. During the rest of this phase, when the CBGG was introduced, Katie would move her chair to the other side of her table without asking.

When the CBGG was withdrawn, a request was put to Ms. Leonard by the researcher to keep Katie in her “CBGG seat” during observations to see if behavior would revert to levels similar to the initial baseline phase. Ms. Leonard agreed to this request. During this withdrawal phase, Katie’s AEB immediately decreased and remained low across the first three data points before increasing toward the end of the phase (*M* = 74.2%, range = 61.1%–90%). During the fourth data point in this phase (data point 12, Figure 2), a new seating plan was put in place for all students and everyone was moved to a different seat. Katie’s increase in AEB during this phase coincided with the seating plan change. Katie’s DB increased in this withdrawal phase, with an increasing trend overall (*M* = 32.8%, range = 15%–45%). No DB data points in this phase overlapped with the preceding CBGG phase. When the CBGG was reintroduced, Katie’s AEB was initially similar to the withdrawal phase, before decreasing substantially for two data points, and increasing again during the final data point (*M* = 84.2%, range = 72.5%–94.7%). Despite the instability, the overall level of AEB was higher than during the preceding withdrawal phase and the overall level was similar to that of the whole class. DB was variable in this phase, remaining higher than the whole class on average. There was a lot of overlap with the preceding withdrawal phase (*M* = 23.5%, range = 5%–42.1%).

The schedule thinning procedure saw Katie’s AEB remain relatively high and stable. With a 3 minute schedule, AEB occurred at a mean rate of 93% (range = 88.9–95%). DB was initially quite low when this adaptation was made, with an upward trend across the three data points (*M* = 10.6%, range = 5%–16.7%). The seating plan changed again, coinciding with the introduction of the 4 minute schedule. AEB remained high and occurred at a mean rate of 91.54% (range = 84.6%–95%). DB remained low and followed a decreasing trend across the phase (*M* = 6.8%, range = 0%–15.4%). Katie was only present for one of the two observations as the schedule was thinned to 5 minutes, and AEB occurred during 73.3% of intervals. This reflects a moderate decrease when compared with the 4 minute schedule. DB was high during this data point (40%; data point 25).

### Effect Sizes

Table 2 provides an overview of the Tau effect sizes. The weighted mean Tau values were large for whole class AEB and DB, for Ellie’s AEB and for Katie’s DB. Effect sizes for Ellie’s DB and Katie’s DB were deemed moderate.

**Table 2.** Tau Effect Sizes Across Phase Changes for the Whole Class and Target Students.

Outcome	Tau A1–B1	Tau A2–B2^a^	Weighted mean Tau
Whole Class			
Disruptive behavior	−0.77	−0.531	−0.67
Academically engaged behavior	0.756	0.762	0.76
Ellie			
Disruptive behavior	−0.674	−0.46	−0.58
Academically engaged behavior	0.674	0.712	0.7
Katie			
Disruptive behavior	−0.775	−0.241	−0.57
Academically engaged behavior	0.756	0.463	0.63

a B2 refers to the second CBGG phases with 2 minutes intervals between points only. Tau values do not take the schedule thinning procedure into account.

### Social Validity

#### Teacher social validity

Teacher feedback collected via self-report on the BIRS (Elliott & Von Brock Treuting, 1991) was positive. Ms. Leonard scored the game 90 out of a possible 90 on the acceptability subscale, responding with “strongly agree” to all statements. On the effectiveness subscale, she scored the game 35 out of a possible 42. The mean rating on this scale was 5 (range = 4–6). The teacher’s rating on the efficiency subscale was 11 out of a possible total of 12. The mean rating on this scale was 5.5 (range = 5–6).

Ms. Leonard was asked to provide additional comments on the schedule thinning element of the game and some general comments. When asked whether she had a preference for any particular version (2, 3, 4, or 5 minutes between points), she stated that the 2 to 3 minutes intervals were “intense.” When asked about her perceptions of effectiveness across the different time intervals, she responded as follows: “*Shorter intervals kept children’s attention on whether they were getting rewards in the beginning but for the long term use [of] 5 min intervals is more manageable*.” She also stated that the 5 minute version of the game was “*easier to implement*.” When asked for additional general comments, Ms. Leonard stated that while the CBGG worked well, sometimes she found it difficult to be consistent with the timetable (i.e., endeavoring to do mathematics class at the same time every day).

#### Student social validity

Nineteen students completed the modified CIRP (Mitchell et al., 2015; Witt & Elliott, 1985). The mean rating across the survey was 7.3 (range = 5–8), indicating a positive perception of the game overall. In additional comments, one student stated there were things his friends didn’t like about the game, like “*when they lose and don't get stamps*.” Another student stated that there were things they did not like about the game and when asked why, responded stating “*When friends distracted me*.” One student who thought the game was not fair expanded, stating that he was “*out of his seat only for a minute*” and that could have cost his team a point.

## Discussion

The current study aimed to evaluate the effectiveness of the CBGG with a lower primary school (senior infant) population and to investigate whether the effectiveness of the game could be sustained when the schedule of reinforcement was thinned. Individual and group behavior was monitored to assert whether students displaying particularly high levels of disruptive behavior responded differently to the game compared to the whole class group. The results of the current study support the efficacy of the CBGG in increasing AEB and decreasing DB in a mainstream, senior infants class. The CBGG with 2 minute intervals between reinforcement opportunities produced significant increases in AEB and decreases in DB across the class across two intervention phases, and the schedule thinning procedure demonstrated that the game could remain effective with longer intervals between reinforcement opportunities. The CBGG was also effective in targeting AEB and DB in two individual target students, although effects were not as strong as for the whole class.

When observing the whole class data, it is evident that the CBGG was effective, producing large weighted mean effect sizes on AEB (0.76) and DB (−0.67). These findings align with previous research which demonstrated the efficacy of the CBGG with young primary school students. For example, Tanol et al. (2010) investigated the CBGG with individual students across two kindergarten classes, finding that it led to reductions in rule violations. In that study, the teachers were responsible for determining the rate of reinforcement and were not prompted to carry out “behavior checks” at fixed intervals making the schedule of reinforcement variable across the 10 minute game session. The present study manipulated the rate of reinforcement delivered by the teacher by establishing schedules. Lynne et al. (2017) also demonstrated that the CBGG could be an effective intervention with a young primary school aged population (a first grade class), but again, the rate of reinforcement appeared to be determined by the teacher. The current study saw the CBGG applied with more structure, similarly to how it was applied in studies by Wright and McCurdy (2012) and Wahl et al. (2016). Wright and McCurdy (2012) demonstrated the efficacy of the CBGG with a kindergarten class on a VI4min schedule and Wahl et al. (2016) produced similar results in four young primary school classes on a VI5min schedule. Although similar, the present study focused on establishing control over behavior with a dense fixed schedule in the initial phases, before thinning the procedure gradually. Wright and McCurdy only implemented the CBGG in one study phase (ABAC design) and Wahl et al., did not incorporate a withdrawal phase. The present study addressed these shortcomings with three attempts to demonstrate an intervention effect (WWC, 2020) before introducing a thinning procedure.

In general, the target students’ levels of AEB and DB increased and decreased respectively when the CBGG was in place compared to baseline/withdrawal phases, but improvements were not as potent as across the whole class group. The CBGG produced large weighted mean effect sizes for AEB and moderate weighted mean effect sizes for DB across both students. The CBGG produced very similar effects on the target students’ DB and the game produced a slightly larger effect on AEB for Ellie compared to Katie. As noted in the results section, Katie’s initial improvements in behavior must be analysed considering the identified confounds related to the seating plan. It was clear that the game appeared most effective for Katie when considering the transition from phase A1 to B1 (i.e., coinciding with the change in seats), rather than between phase A2 and B2. The results demonstrate how individual student behavior can be sensitive to small changes in the environment such as the seating plan, whereas the whole class behavior can remain quite stable under these changes.

The findings from the two individual students lend support to the idea that although an intervention may appear to be highly effective across a whole class group, individual students may not respond as positively. For example, during the initial baseline phase, Ellie’s DB was similar to that of the whole class, however across the whole class, there was a mean decrease of 21.77% when the CBGG was introduced, compared to a smaller, 14.48% decrease for Ellie. Previous studies have similarly identified non-responders to the GBG and CBGG. Donaldson et al. (2017) collected data on 12 individual students as part of an evaluation of the GBG with individuals and found that 3 of the 12 students did not respond positively to the GBG. They could therefore be potentially classified as “non-responders” and be referred for more intensive behavioral support. In another study, Bohan and Smyth (2022) found that individual target students responded well to the CBGG and that at times the game brought their behavior more in line with the behavior of the class group. During some sessions however, one target student in that study engaged with high levels of DB and low levels of AEB even when the intervention was in place. In the present study, neither target student was a total non-responder and both benefited in some way from the CBGG.

Results from the schedule thinning phase support the idea that schedule thinning is a potential solution to lessening the workload for the teacher during CBGG implementation, such that over time, teachers may be able to systematically decrease the amount of behavior checks conducted during the game. Although the CBGG has been applied with variable schedules (Wahl et al., 2016; Wright & McCurdy, 2012), dense schedules (Ford et al., 2022), and teacher determined schedules (Tanol et al., 2010), no previous study had looked at beginning with a dense schedule and thinning it over time. Other research has demonstrated the efficacy of schedule thinning during DRO procedures for other behaviors (e.g., Bergstrom et al., 2011). Future research should perhaps consider examining within session schedule thinning. This would involve the teacher playing the game with a dense schedule initially (e.g., 2 minute intervals) and progressing to larger intervals within the same session/day. This may be a potential solution for teachers who are concerned with behavior reverting quickly to baseline levels when the game is withdrawn (e.g., Donaldson et al., 2015).

### Limitations

The results of the current study must be considered in light of a number of limitations. The change in seating plan on occasion throughout the study (individual student changes and whole class changes) was a confounding variable which could not be avoided and which may be considered a limitation. Importantly however, seating plan changes are a regular occurrence in school classrooms, with teachers often changing their seating plan monthly, therefore the changes reflect real classroom practices. Another limitation was that treatment integrity, although relatively high on average, was low on occasion throughout the study, particularly during the very last data point. This limits conclusions which can be drawn for the CBGG with 5 minute intervals between reinforcement opportunities. Another limitation relates to the method of choosing the target students. The students were chosen based on teacher evaluation of the class. A more systematic method, such as collecting baseline data on all students, may ensure that students are chosen more objectively for individual monitoring. Finally, a schedule thinning procedure was used here and started with 2 minute intervals between opportunities for points and progressed to 5 minute intervals. It is possible that the class may have responded well to a thin schedule of reinforcement in the first instance. Future research could perhaps be more objective in choosing a starting point for intervals by probing the game with differing interval lengths or calculating the mean inter-response time between incidences of DB of students engaging in the most disruptive behavior. Furthermore, the schedules used here were similar to those used in other studies and therefore a greater contribution to knowledge may be made by thinning schedules even further. Nevertheless, the current study is the first to examine schedule thinning during the CBGG, and has demonstrated that the game can be effective with successively thinner schedules of reinforcement over time. This paves the way for much further research with varying schedules lengths.

### Implications for Future Research and Practice

The current study provides further evidence for the use of the CBGG in primary school classrooms, particularly with younger classes. It is also one of few studies demonstrating the effectiveness of the CBGG in an Irish primary school setting, building upon other work in Irish (Bohan & Smyth, 2022; Bohan et al., 2021, 2022) and international settings (Groves & Austin, 2020; Wahl et al., 2016; Wright & McCurdy, 2012). Although the evidence is preliminary, taken with other evidence, Irish teachers, and other teachers in lower primary school contexts, may consider this game for use in their classrooms. The study has demonstrated the game’s efficacy across a whole class group but has also demonstrated that not all individual students respond in the same way to the CBGG. This suggests that teachers should be mindful of (a) students who do not respond as well to class wide interventions and (b) total non-responders. Ellie and Katie would not be classed as total non-responders in this study and the CBGG did have some positive effects for each of them respectively, however, future research may investigate ways to intervene when individuals do not respond as well as the whole group. The evidence here for schedule thinning as part of the CBGG is promising and provides a starting point for further research on the concept. Future research may evaluate whether the CBGG is effective initially with a dense schedule, and then prolong the game with a thinned schedule during the same session. This may provide a solution for teachers who are concerned that disruptive behavior becomes an issue immediately after game cessation. It may also be a worthwhile avenue to examine if the CBGG can be effective with even thinner schedules of reinforcement beyond the 5 minutes examined in the current study.

## Conclusion

Overall, the current study has provided further good quality evidence for the CBGG with a lower primary population. The concept of schedule thinning as part of the CBGG has been introduced, and this serves as a useful starting point for further examination in different contexts. Individual students responded well but differently to the whole class during this iteration of the CBGG. Therefore, the benefits of collecting data on individual students as well as the whole class group cannot be ignored in research on group-based interventions, such as group contingencies.

## Supplemental Material

sj-pdf-1-bmo-10.1177_01454455221129993 – Supplemental material for The Effect of Schedule Thinning on Student Behavior During the Caught Being Good Game Click here for additional data file. Supplemental material, sj-pdf-1-bmo-10.1177_01454455221129993 for The Effect of Schedule Thinning on Student Behavior During the Caught Being Good Game by Clare Bohan and Sinéad Smyth in Behavior Modification
