# Supplementary material for: The Effect of Schedule Thinning on Student Behavior During the Caught Being Good Game
Source: Behav Modif. 2022 Nov 13;47(3):644–69. doi: 10.1177/01454455221129993 (PMC10150262; doi:10.1177/01454455221129993)
Supplement: sj-pdf-1-bmo-10.1177_01454455221129993 – Supplemental material for The Effect of Schedule Thinning on Student Behavior During the Caught Being Good Game [file sj-pdf-1-bmo-10.1177_01454455221129993.pdf]

# The Caught Being Good Game Training

January 2020

# Why are we doing this study?

- Studying for a PhD in Psychology- focus on behaviour management
- Especially interested in positive behaviour support and managing behaviour through proactive strategies

# How does the CBGG work?

- Based on the premise of **positive reinforcement** and **group contingencies**
- **Interdependent Group Contingency:** Every member of a group/team must meet a certain standard for a prize/reinforcement to be delivered
- **Example:** If every student in the class completes their homework on a Thursday night, the whole class get some free time during Friday’s class. If even one student doesn’t do their homework, no one gets free time.

# How does the CBGG work?

- The CBGG is a game-based interdependent group contingency
- Steps:
  - Class divided into teams (2 or more- will be tables in this class).
  - Set of positively phrased classroom expectations displayed and explained to students.
  - Students are told that they can earn points for their team by following these expectations and that teams reaching X amount of points by the end of the game (20 mins) will get a prize.
  - Teacher sets a timer to go off at different times during class. When this timer goes off, the teacher scans the room and teams on which all members are following the classroom expectations gain a point.
  - If even one team member is breaking a rule, the team does not get a point.
  - Teams win if they reach a certain criterion of points by the end of the game.

# Research Project

- We have a number of aims with this research project:
  - Is the CBGG effective with primary school populations in Dublin?
  - Is the game effective with both class groups and individuals?
  - Can the game be modified to give out less points (thin the reinforcement schedule) and still be effective?

# Timeline

Baseline= "Business as usual"

- Week 1: Baseline
- Week 2: CBGG with points every 2 min
- Week 3: Baseline
- Week 4: CBGG with points every 2 min
- Week 5: CBGG with points every 3 min- 4 min
- Week 6: CBGG with points every 4 min-5 min
- Week 7: CBGG with points every 5 min- 6 min

**Note: Phases may last slightly over one week depending on school term/days off etc.**

# Materials (Younger students): Daily Scoreboard

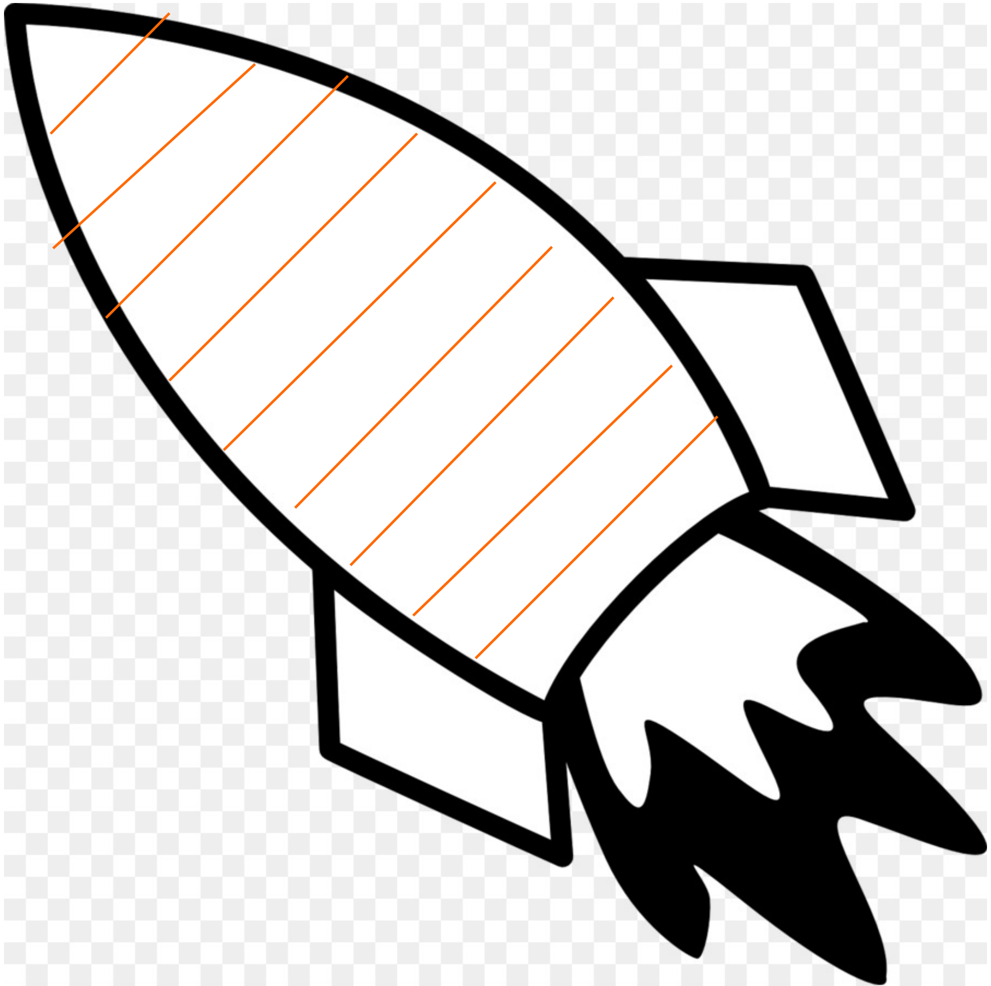

# Materials: Class Rules

- Look at and Listen to your teacher
- Do your best at your work
- Respect your friends & let them do their work
- Stay in your seat
- Hands up and wait for your teacher

# Timer: When to Award Points

- I will set up a Fitbit which will vibrate every two minutes.
- When it vibrates, that's your cue to carry out a behaviour check and award points.
- If you are busy when the vibration occurs, don't worry, just try and carry out your check before the next vibration.
- Press the button on the Fitbit twice to dismiss the alarm. If you only press it once, it goes into 'snooze mode' and will vibrate again in ten minutes.

# Materials: Timer Set Up

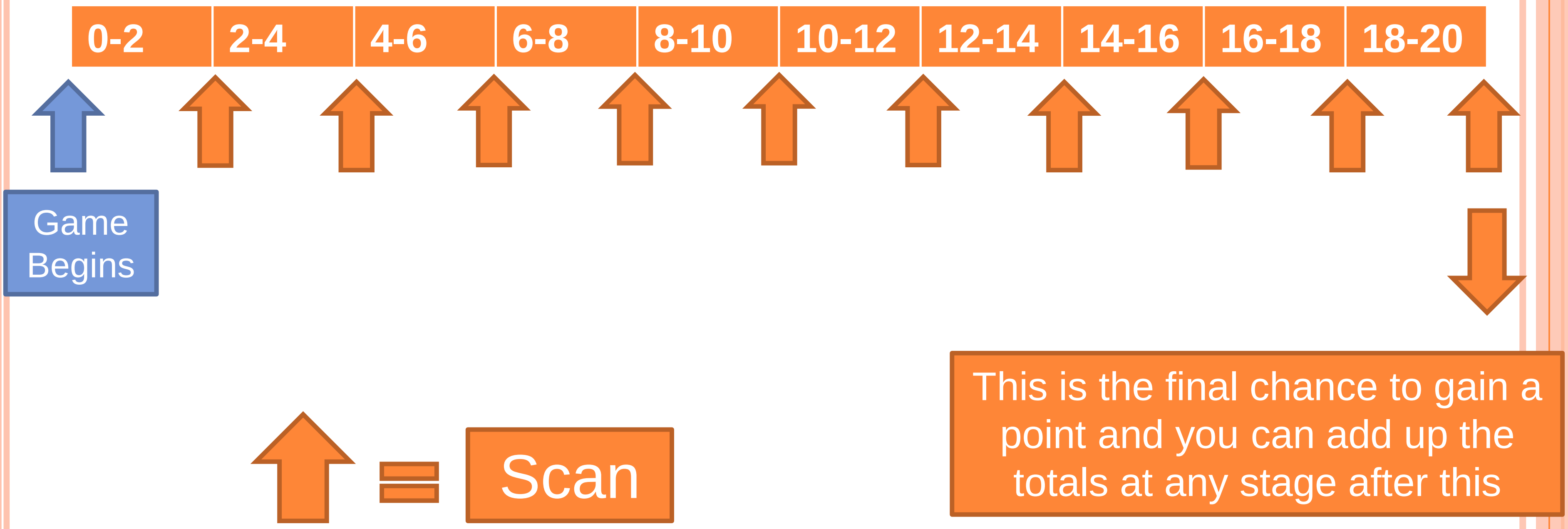

# Materials: Prizes

- We need to come up with a list- Ideas?
- Could do treasure chest vs stamps vs stickers and team with most points gets to vote

# Materials: Teacher Procedure and Checklist

- Procedure is given to you as a guide in putting the game in place
- You will have an 11 step checklist which I will be filling in daily during CBGG phases. If this checklist is not being followed to 80% accuracy, I will contact you and let you know.

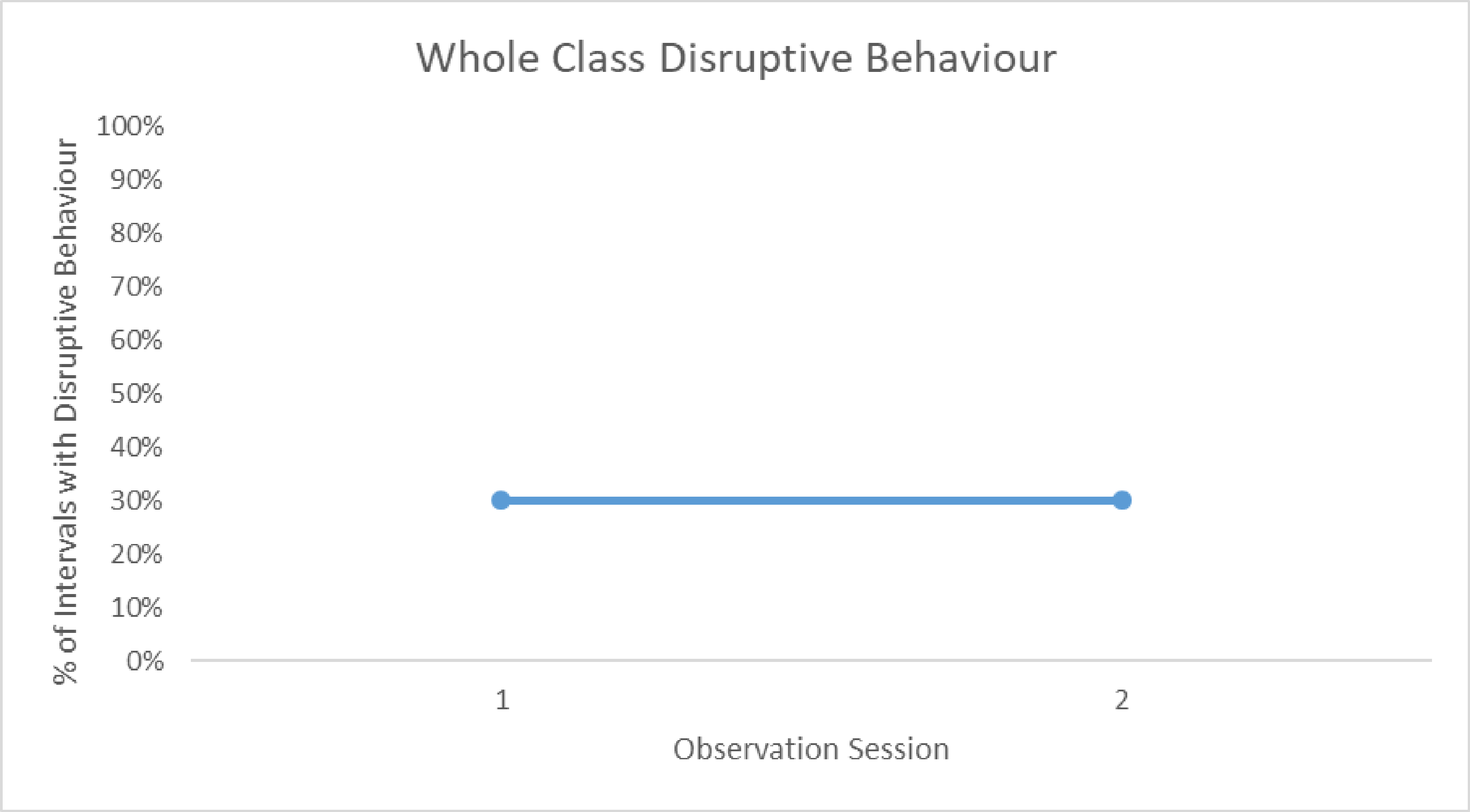


**Mean Rate of Disruptive Behaviour: 30%**

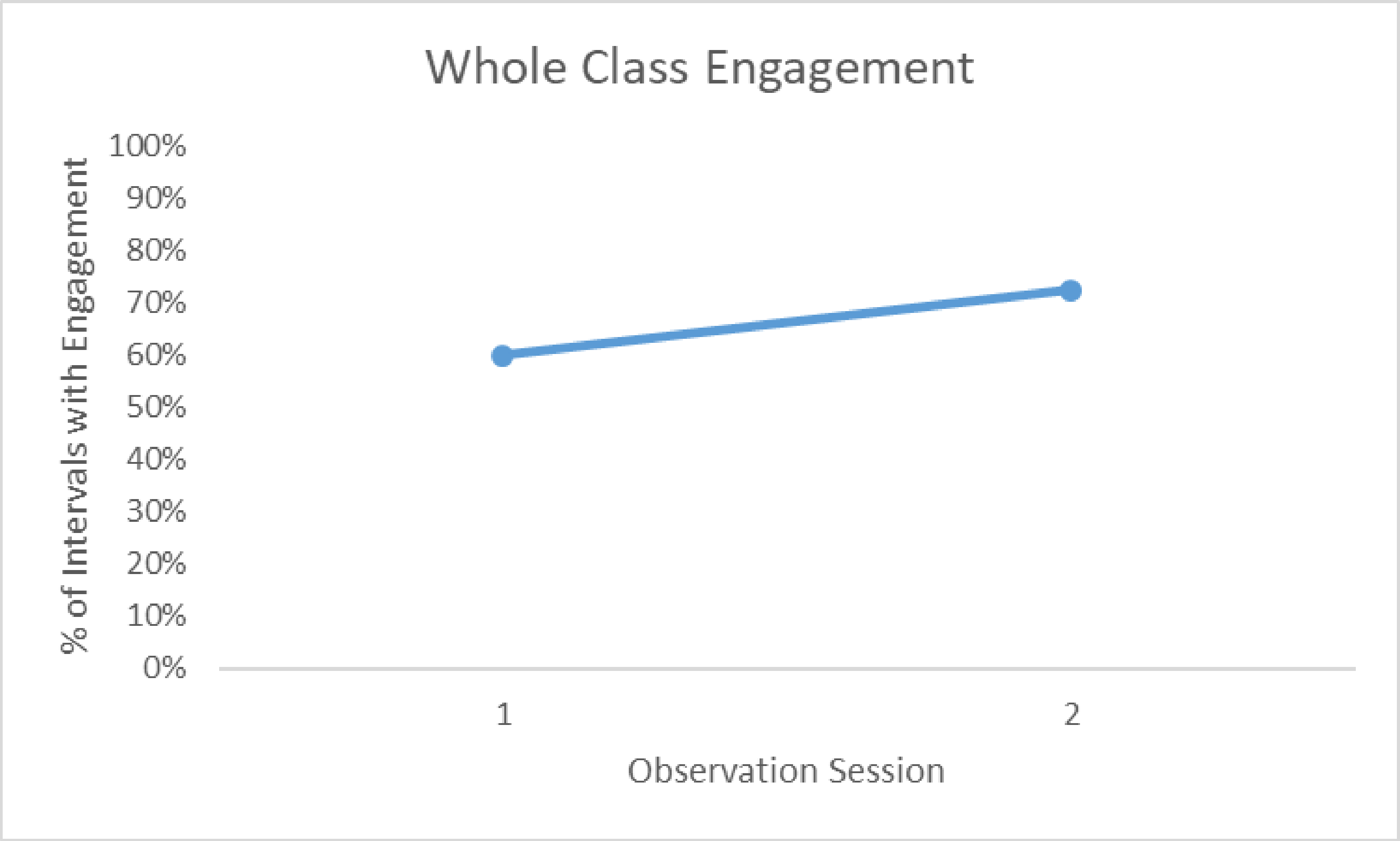


**Mean Rate of Engagement: 66.25%**

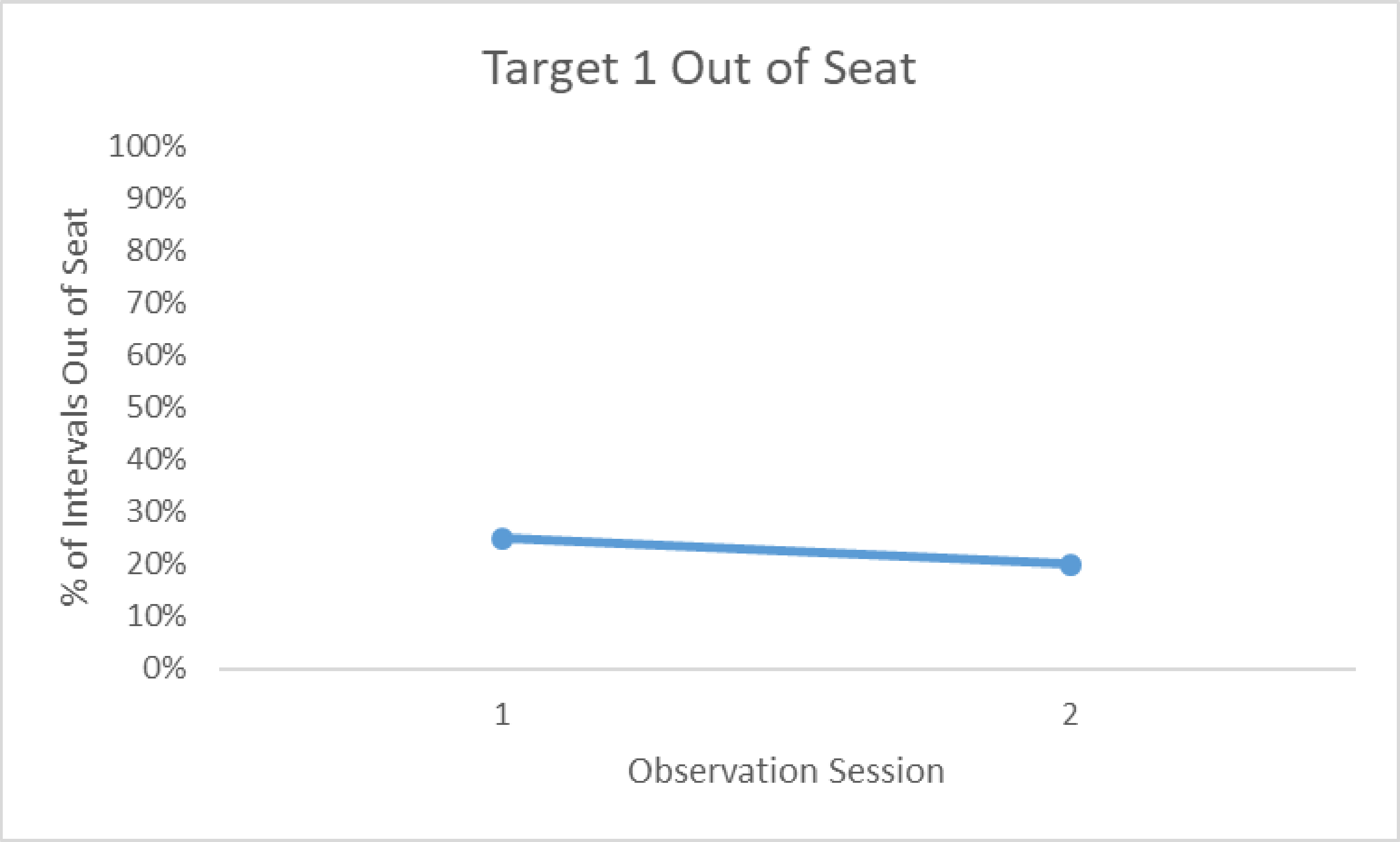


**Mean Rate of Out of Seat: 22.5%**

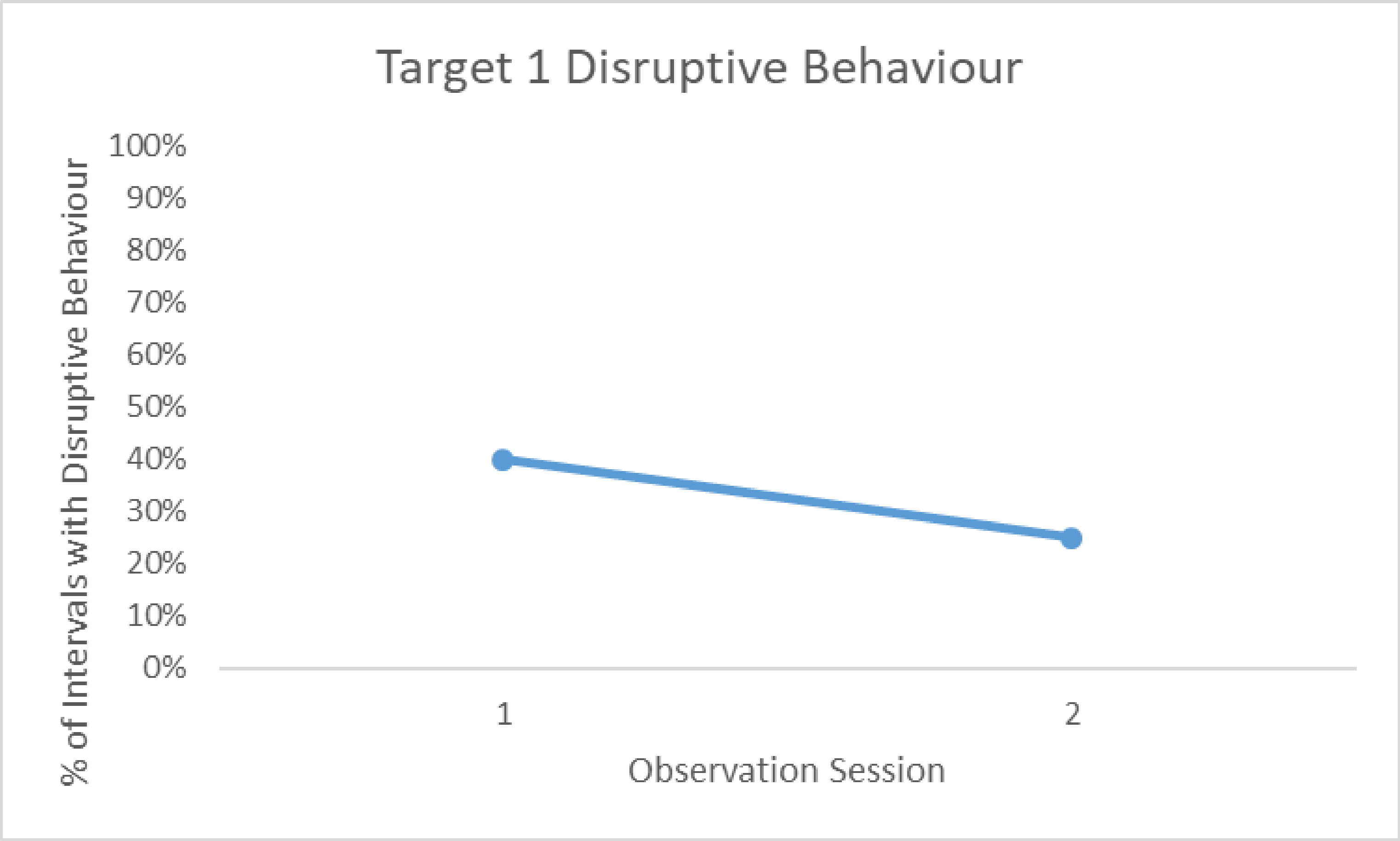


**Mean Rate of Disruptive Behaviour: 32.5%**

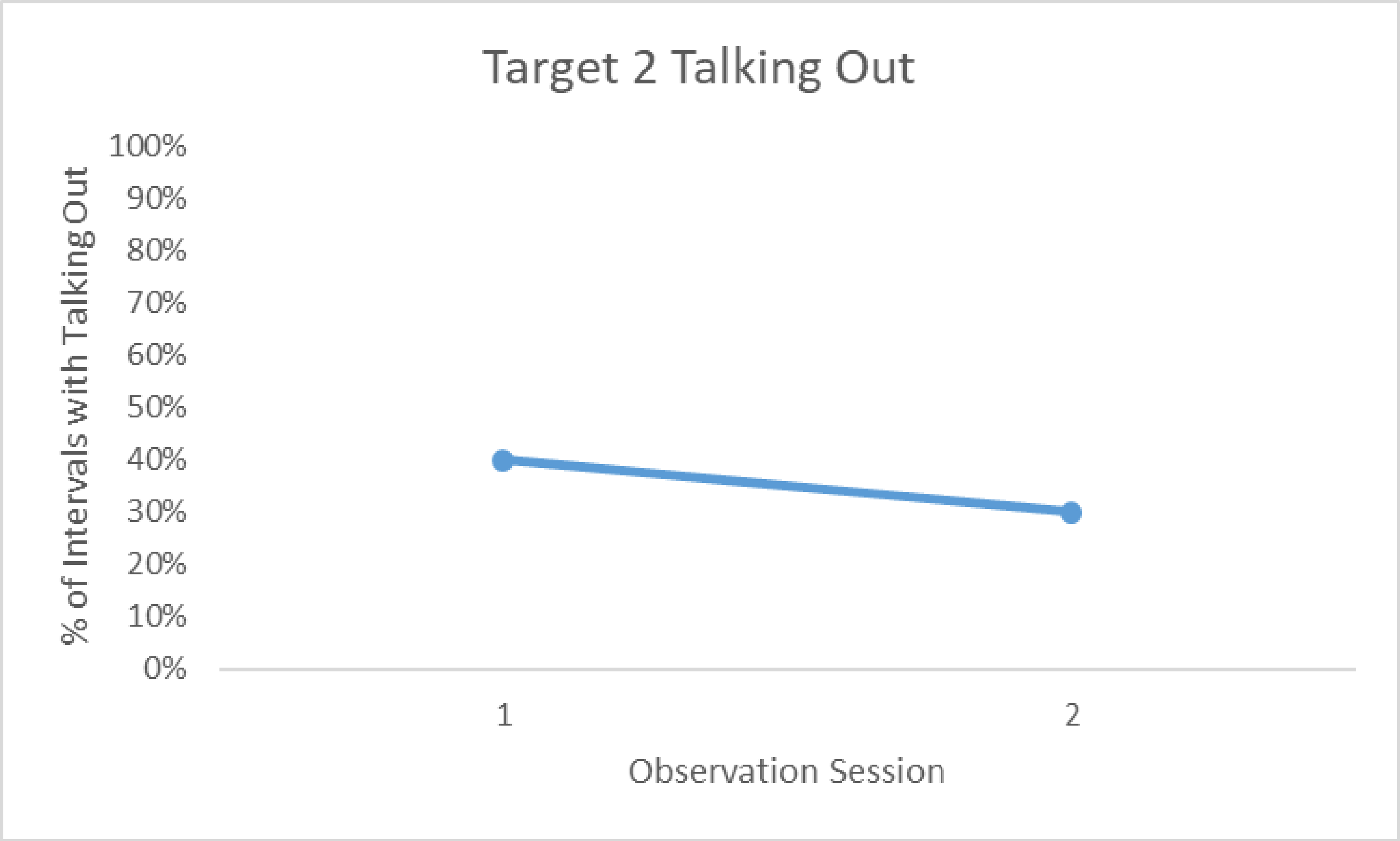


**Mean Rate of Talking Out: 35%**

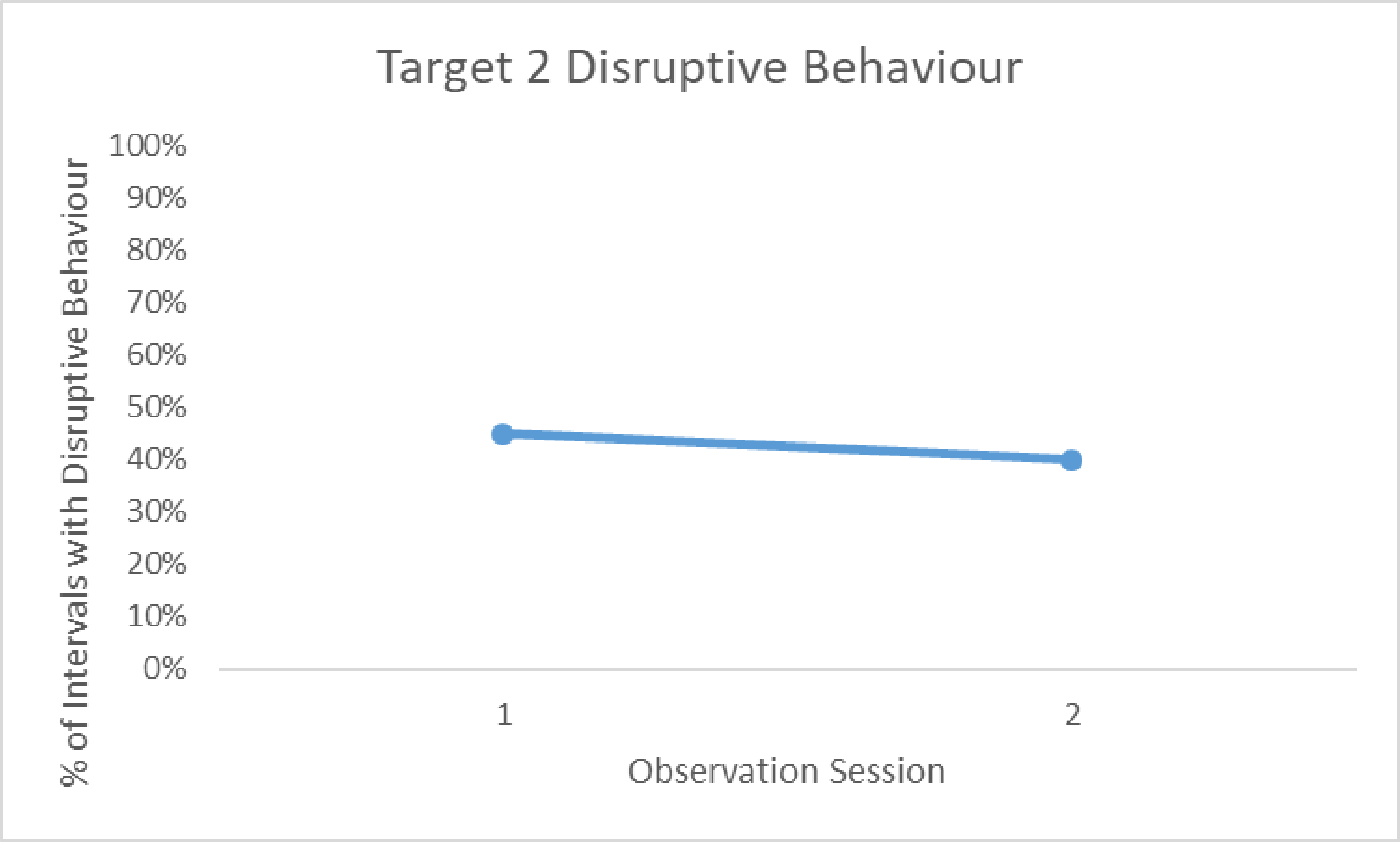


**Mean Rate of Disruptive Behaviour: 42.5%**
